# Orificial Surgery

**Published:** 1895-07

**Authors:** 


### ORIFICIAL SURGERY.

Orificial surgery! The last and most brilliant in the long list of medical humbugs which the century has furnished, such as Thomsonianism, physio-medical tomfoolery, vitapathy, magnetic healing, Christian Science, etc. Time's noblest offspring is the last.

The alleged achievements of the orificial surgeon, as set forth in an orificial exchange, are little short of the marvelous, and force the conclusion that the fool-killer has abandoned his occupation. We read of a late case of "phthisis cured by the operation for laceration of the cervix." A case of chronic eczema of the hands was "cured by stretching the rectum." Another case of eczema was treated by "clipping irritated points at the various outlets of the body." This seems vague but heroic. And so on. For all the ills that afflict the flesh the blame is laid at the door of one, or some, of the orifices of the body, from the puncta lachrymalis to the meatus urinarius, and this, or they, must needs be opened and stretched by the most approved orificial methods. A worse species of quackery was never invented. One orificial gentleman thinks that a "conservative orificial surgeon should be connected with each of our State hospitals for the insane." An exchange says this idea is an excellent one, if he be connected in the proper capacity—as patient rather than as surgeon.

